# The role of oxidative stress in the pathogenesis of infections with coronaviruses

**DOI:** 10.3389/fmicb.2022.1111930

**Published:** 2023-01-13

**Authors:** Chandrima Gain, Sihyeong Song, Tyler Angtuaco, Sandro Satta, Theodoros Kelesidis

**Affiliations:** Department of Medicine, Division of Infectious Diseases, University of California, Los Angeles, Los Angeles, CA, United States

**Keywords:** SARS-CoV-2, coronavirus, inflammation, oxidative stress, tissue damage, apoptosis

## Abstract

Coronaviruses can cause serious respiratory tract infections and may also impact other end organs such as the central nervous system, the lung and the heart. The coronavirus disease 2019 (COVID-19) has had a devastating impact on humanity. Understanding the mechanisms that contribute to the pathogenesis of coronavirus infections, will set the foundation for development of new treatments to attenuate the impact of infections with coronaviruses on host cells and tissues. During infection of host cells, coronaviruses trigger an imbalance between increased production of reactive oxygen species (ROS) and reduced antioxidant host responses that leads to increased redox stress. Subsequently, increased redox stress contributes to reduced antiviral host responses and increased virus-induced inflammation and apoptosis that ultimately drive cell and tissue damage and end organ disease. However, there is limited understanding how different coronaviruses including SARS-CoV-2, manipulate cellular machinery that drives redox responses. This review aims to elucidate the redox mechanisms involved in the replication of coronaviruses and associated inflammation, apoptotic pathways, autoimmunity, vascular dysfunction and tissue damage that collectively contribute to multiorgan damage.

## Introduction

The coronavirus disease 2019 (COVID-19) has had a devastating impact on humanity. Coronaviruses can cause serious respiratory tract infections and may impact other end organs such as the central nervous system. Coronaviruses are enveloped single-stranded positive-sense RNA viruses named after their crown-like appearance of their spike proteins on their surface (Singhal, 2020). To date, there has been seven human coronaviruses (HCoVs) identified: severe acute respiratory syndrome coronavirus (SARS-CoV-2), SARS-CoV, Middle East respiratory syndrome coronavirus (MERS-CoV), Human coronavirus 229E (HCoV-229E), HCoV-OC43, HCoV-NL63, and HKU-1. Four of them including HCoV-OC43, HCoV-NL63, HCoV-229E, and HKU-1, typically trigger only mild respiratory illnesses in humans. On the other hand, SARS-CoV-2, SARS and MERS are known to cause more severe illness, acute respiratory distress syndrome (ARDS) or multi-organ dysfunction, especially in aged people with comorbidities (Li et al., 2021a). Understanding the mechanisms that contribute to the pathogenesis of coronavirus infections, will set the foundation for development of new treatments to attenuate the impact of coronaviruses on host cells and tissues. However, there is limited understanding how different coronaviruses including SARS-CoV-2, manipulate cellular machinery to drive host cell responses.

**Figure 1:**
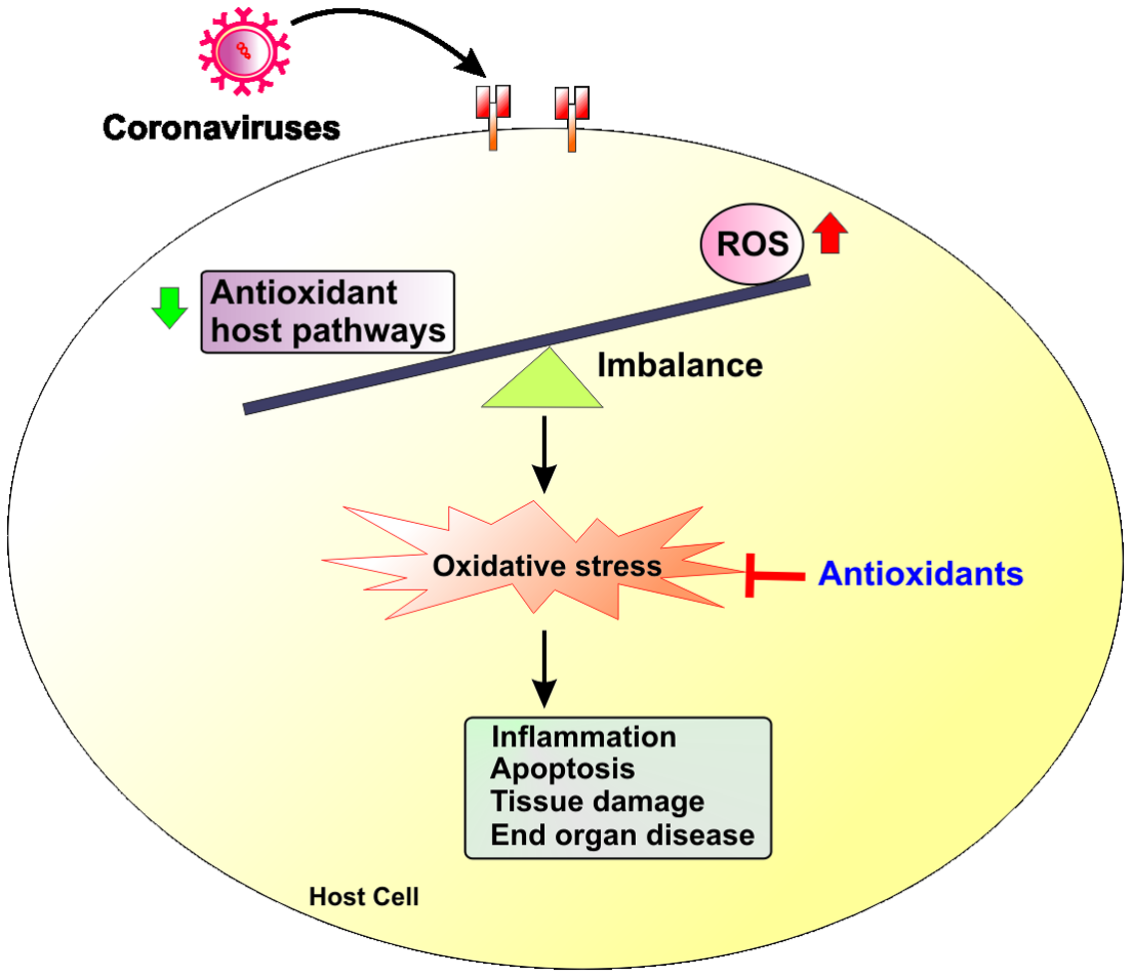
Redox imbalance in coronavirus infections. Coronavirus infection triggers an imbalance between increased production of reactive oxygen species (ROS) and reduced antioxidant host responses that leads to increased redox stress in the host cell. Increased redox stress induces inflammation, apoptosis and ultimately tissue damage and end organ disease.

Emerging evidence suggests that human diseases including viral infections often disrupt the host natural balance between increased production of reactive oxygen species (ROS) and reduced antioxidant host responses that collectively increases redox stress (Amini et al., 2022; Figure 1). ROS are free radical and nonradical byproducts of metabolic processes in organelles such as plasma and nuclear membranes, the mitochondria, peroxisomes and the endoplasmic reticulum (ER; Reshi et al., 2014). ROS are necessary for cellular processes like mitochondrial energy production, host defense, cellular signaling, and the regulation of gene expression. Mitochondria are the main location of production of ROS (mito-ROS) during energy production. Increased ROS during viral infections have not only detrimental impact on the cells and tissues but are also important for antiviral immune function (Yang et al., 2007; Finkel, 2011) during viral infections like influenza (To et al., 2014), respiratory syncytial virus (RSV; Fink et al., 2008) and rhinoviruses (Kaul et al., 2000; Fink et al., 2008).

However, an excess of ROS can damage cellular components including lipids, proteins, and DNA, alter immune functions, inflammatory responses and induce organ and tissue dysfunction (Preiser, 2012; Reshi et al., 2014; Labarrere and Kassab, 2022). Indeed, several studies have shown that oxidative stress contributes to the pathogenesis of respiratory viral infections (Khomich et al., 2018), influenza and RSV. Increased oxidative stress in severe COVID-19 contributes to inflammation, endothelial cell dysfunction, thrombosis that can lead to multiorgan damage (Li et al., 2021a; Alam and Czajkowsky, 2022). Oxidative stress, induced by coronavirus, also interferes with inflammatory pathways that may lead to more long-lasting tissue damage. However, there is limited understanding how different coronaviruses including SARS-CoV-2, manipulate cellular machinery that drives redox responses.

In this review, we summarize the scientific evidence regarding the cellular and molecular pathways modulated by oxidative stress that are implicated in the pathogenesis of coronavirus infections. We specifically review the role of redox pathways in major pathophysiological underpinnings that contribute to cell and tissue damage in coronavirus infection: (1) virus replication, (2) virus-associated inflammation, (3) virus-associated apoptosis, (4) redox-related end organ disease. We review the scientific evidence related to these redox pathways, separately for SARS-CoV-2 versus all the other coronaviruses [SARS-CoV, MERS, respiratory coronaviruses and other coronaviruses used to model SARS-CoV-2 infection such as the murine hepatitis virus (MHV)]. Finally, we discuss the relevance of these redox pathways with regards to acute severe COVID-19 and Post-Acute Sequelae of SARS-CoV-2 infection (PASC) and potential antioxidant treatments.

## Redox mechanisms that regulate replication of coronaviruses

**Figure 2:**
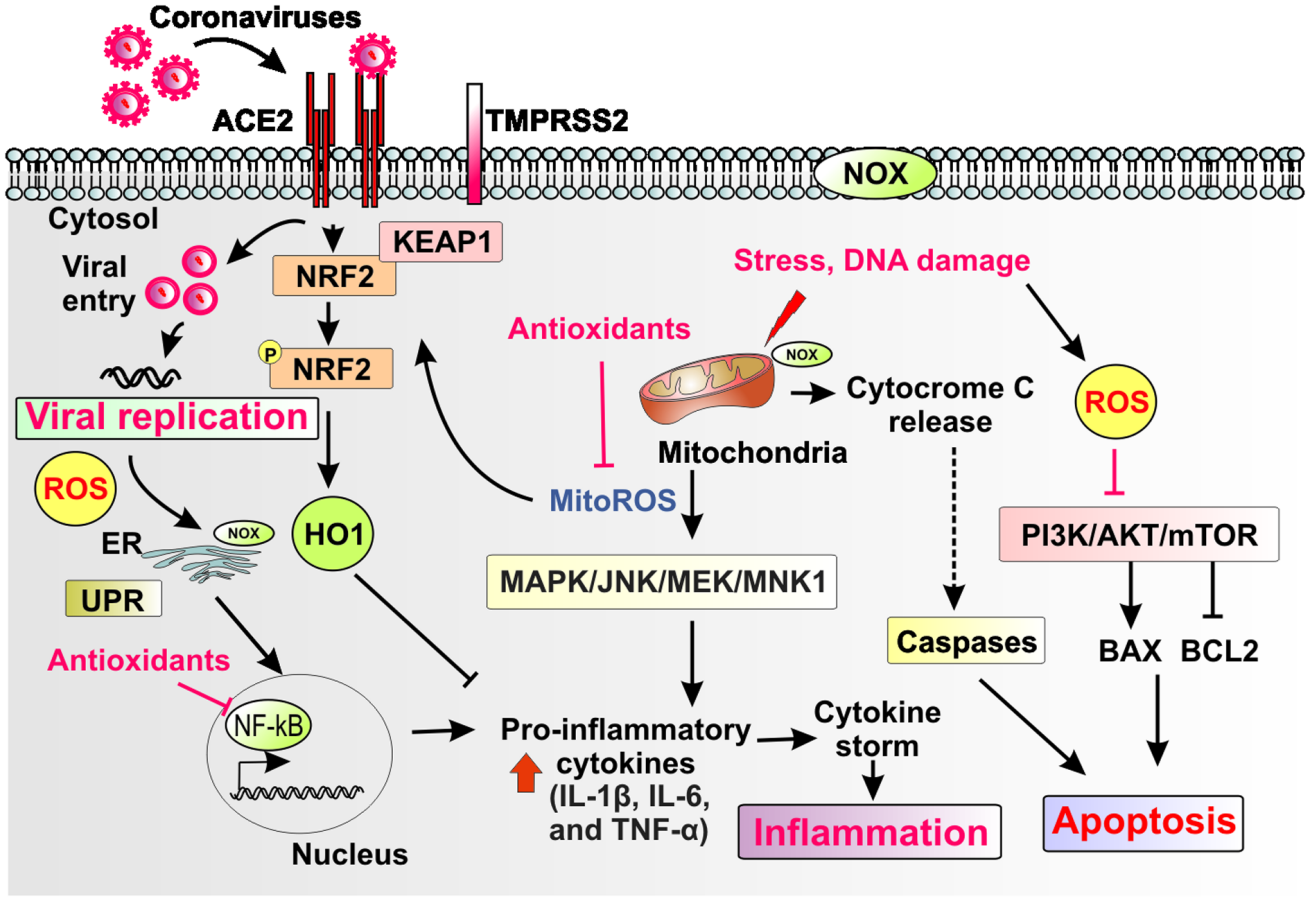
Schematic representation of redox pathways that contribute to viral replication, inflammation, and apoptosis during coronavirus infection. Coronaviruses bind to the ACE2 receptor and replicate through host proteases such as TMPRSS2 and by hijacking cytosolic cellular machinery such as the mitochondria and the endoplasmic reticulum (ER), which engages the unfolded protein response (UPR). The plasma membrane, the ER and mitochondria harbor different isoforms of the NADPH oxidase (NOX) enzyme. Coronaviruses induce cellular oxidative stress with generation of reactive oxygen species (ROS) and mitochondrial ROS (mito-ROS) and impairment of stress-inducible, antioxidant, anti-inflammatory and antiviral responses such as the Nrf2 pathway and other key downstream mediators such as Heme oxygenase-1 (HO-1). Mito-ROS induce downstream signaling pathways such as MAPK, JNK, MEK/MNK1 that induce both viral replication and proinflammatory pathways such as induction of cytokines (e.g., IL-1b, IL-6, and TNF-a). Mito-ROS, ROS and ER stress response induce the proinflammatory pathway NF-κB. ROS and mito-ROS also induce apoptosis through alterations in apoptotic pathways such as PI3K/AKT, mTOR and induction of mitochondrial apoptosis. Collectively, redox mediated pathways that drive viral replication, inflammation and apoptosis contribute to cell and tissue damage that drive end organ disease in coronavirus infection. Endogenous antioxidant host pathways and exogenous therapeutic antioxidants could attenuate redox mediated pathways that drive pathogenesis of coronavirus infections.

Several redox mechanisms can regulate both viral entry and cytosolic replication of coronaviruses (Figure 2; Table 1; Wang and Zhang, 1999; Kulisz et al., 2002; Halestrap et al., 2004; Mizutani et al., 2004; Emerling et al., 2005; Kefaloyianni et al., 2006; Doughan et al., 2008; Lucas et al., 2008; Cho et al., 2009; Garrido and Griendling, 2009; Hosakote et al., 2009; Jamaluddin et al., 2009; Wosniak et al., 2009; de Wilde et al., 2011; Kesic et al., 2011; Xia et al., 2011; Kosmider et al., 2012; Yamada et al., 2012; Kim et al., 2012b; Lee et al., 2013; Nguyen Dinh Cat et al., 2013; Komaravelli and Casola, 2014; Hyser and Estes, 2015; Kindrachuk et al., 2015; Komaravelli et al., 2015; Paszti-Gere et al., 2015; Shirihai et al., 2015; Simon et al., 2015; Demers-Lamarche et al., 2016; Kau et al., 2016; Morris et al., 2016; Zhang et al., 2016; Daiber et al., 2017; Trempolec et al., 2017; Khomich et al., 2018; Tu et al., 2019; Olagnier et al., 2020; Tao et al., 2020; Verdecchia et al., 2020; Herengt et al., 2021; Moghimi et al., 2021; Youn et al., 2021).

**Table 1:** Redox mechanisms that regulate replication of coronaviruses.

Mediators	Effect on redox balance	References
Redox mechanisms that may regulate viral entry of coronaviruses		
Bidirectional cross talk between virus and the ACE2-AngII (ligand of ACE2)-NOX axis	↑Ang II →↑ activation of Nox4	Doughan et al. (2008), Garrido and Griendling (2009), Wosniak et al. (2009), Xia et al. (2011), Kim et al. (2012b), Lee et al. (2013), Nguyen Dinh Cat et al. (2013), Daiber et al. (2017), Verdecchia et al. (2020)
	↑ACE2 → ↓ NOX	
	Virus ↓ ACE2 →↑ NOX	
	Bidirectional crosstalk between virus, mitochondria and NOX	
TMPRSS2 (host protease essential for replication of coronavirus)	No solid evidence to support role of redox stress in TMPRSS2 regulation but excess redox stress may alter distribution pattern of TMPRSS in epithelial cells	Lucas et al. (2008), Paszti-Gere et al. (2015), Kau et al. (2016), Moghimi et al. (2021), Youn et al. (2021)
Mito-ROS	↑ vacuole formation through AAK activation	Demers-Lamarche et al. (2016), Morris et al. (2016)
	Alters membrane lipid-based cellular signaling	
Redox mechanisms regulating cytoplasmic replication of coronaviruses		
Mito-ROS	Regulate ER stress and unfolded protein response	Wang and Zhang (1999), Kulisz et al. (2002), Halestrap et al. (2004), Mizutani et al. (2004), Emerling et al. (2005), Kefaloyianni et al. (2006), Jamaluddin et al. (2009), de Wilde et al. (2011), Hyser and Estes (2015), Kindrachuk et al. (2015), Shirihai et al. (2015), Zhang et al. (2016), Trempolec et al. (2017), Tao et al. (2020)
	Regulate Ca^2+^ signaling systems	
	↑ MPTP	
	Regulate mitophagy (protein misfolding, depolarization of mitochondria)	
	↑ MEK, MNK1, MAPK →↑ viral protein synthesis	
	Regulate interferon host responses	
	↑ Nrf2 pathway	
Keap1-Nrf2-ARE pathway	ROS ↑ antioxidant gene expression, →↑ HO-1, NQo-1, SOD, glutathione derived molecules catalase, peroxiredoxins, glutathione peroxidases Respiratory viruses ↓ Nrf2	Cho et al. (2009), Hosakote et al. (2009), Kesic et al. (2011), Yamada et al. (2012), Kosmider et al. (2012), Komaravelli and Casola (2014), Komaravelli et al. (2015), Simon et al. (2015), Khomich et al. (2018), Tu et al. (2019), Olagnier et al. (2020), Herengt et al. (2021)
	↑ stress-inducible, anti-inflammatory, antiviral responses	
	↑ antiviral HO-1	
	↑ antiviral immunity	
	Mediates pathogenesis and tissue damage of many viral infections, including HIV, RSV, Influenza, SARS-CoV-2	
	↓ apoptosis that regulates viral replication (cell death and release of virions)	

Abbreviations: AAK, adaptor-associated kinase; ACE2, Angiotensin-converting enzyme 2; AngII, Angiotensin II; ARE, antioxidant response element; Ca^2+^, Calcium (II) ion; ER, endoplasmic reticulum; HIV, human immunodeficiency virus; HO-1, Heme oxygenase 1; Keap1, Kelch-like ECH-associated protein 1; MAPK, mitogen-activated protein kinase; MEK, Mitogen-activated protein kinase; Mito-ROS, Mitochondrial reactive oxygen species; Mnk1, mitogen-activated protein kinase (MAPK) interacting protein kinase 1; mPTP, mitochondrial permeability transition pore; NOX, nicotinamide adenine dinucleotide phosphate (NADPH) oxidase; Nrf2, nuclear factor erythroid 2–related factor 2; NQo-1, NAD(P)H quinone oxidoreductase; RSV, Respiratory Syncytial Virus; SOD, Superoxide dismutase; SARS-CoV-2, severe acute respiratory syndrome coronavirus 2; TMPRSS2, Transmembrane serine protease 2; UPR, unfolded protein response.

### Redox mechanisms that regulate virus entry of coronaviruses

The spike S proteins on the surface of coronaviruses are responsible to their attachment to host receptors in airway epithelial cells such as the angiotensin-converting enzyme 2 (ACE2) receptors that interact with host cell proteases, such as transmembrane protease serine 2 (TMPRSS2; Hamming et al., 2004; Irigoyen et al., 2016; Lukassen et al., 2020; Xu et al., 2020). While many coronaviruses utilize peptidases, such as ACE2, dipeptidyl peptidase 4, aminopeptidase N, as their cellular receptors, SARS-CoV, SARS-CoV-2 and HCoV-NL63 utilize ACE2 as their receptors thus disrupting the renin-angiotensin system (Verdecchia et al., 2020).

ACE2, a peptidase that exists on the cell surfaces of most organs (Hamming et al., 2004), is one of the most crucial key players in induction of redox stress (Shatizadeh Malekshahi et al., 2022). Angiotensin II (AngII), the ligand of ACE2, is a potent activator of nicotinamide adenine dinucleotide phosphate (NADPH) oxidase and an inducer of ROS production in the vasculature, kidney and brain (Garrido and Griendling, 2009). Typically, ACE2 helps avert NAPDH oxidase activity by converting Ang II into angiotensin 1–7, thereby reducing ROS levels; Ang II stimulates NAPDH oxidase. ACE2 overexpression has been shown to reduce ROS, and ACE2 deficiency has been shown to induce oxidative stress (Xia et al., 2011; Pena Silva et al., 2012). The complex cross-talk between ACE2 and redox pathways is further emphasized by a possible bidirectional redox regulation of ACE2 levels. High ACE2 activity may reduce redox stress but vice versa high redox stress may regulate ACE2 activity. *In vitro* studies showed that NOX-driven ROS may reduce ACE2 in vascular smooth muscle cells (Lavrentyev and Malik, 2009). Consistent with this evidence, independent *in vitro* studies demonstrated that Ang II-induced activation of mitochondrial Nox4 is an important endogenous source of ROS and is related to cell survival in kidney epithelial cells (Kim et al., 2012b). The crosstalk between NOX and ACE2 has also been shown *in vivo* in mouse models of disease and increased levels of ACE2 are generally associated with reduced oxidative stress in mammalian cells (Xia et al., 2011).

Angiotensin II is often upregulated in viral infections (Doughan et al., 2008; Wosniak et al., 2009; Lee et al., 2013; Daiber et al., 2017). However, when cells are infected with coronavirus, there is a reduction of ACE2 receptors on the cell surface and this results in an increase of Ang II which binds to ACE1 and increases ROS levels through NADPH oxidase (Nguyen Dinh Cat et al., 2013). Experimental studies have demonstrated that *in vitro* exposure to S protein induces excessive oxidative stress in endothelial cells, which is mediated specifically by activation of NADPH oxidase isoform 2 (NOX2), but not NOX1 or NOX4 (Youn et al., 2021). However, it is unclear if there is bidirectional link between ACE2 levels and increased redox cellular pathways in the setting of SARS-CoV-2-induced ACE2 downregulation in airway epithelial cells.

TMPRSS2 is expressed in both the cytoplasm as well as in the cell membrane in epithelial cells (Lucas et al., 2008). *In vitro* studies with porcine intestinal epithelial cells have shown that acute excessive oxidative stress induces altered distribution pattern of TMPRSS2 and relocalized transmembrane serine protease activity that may contribute to weakening of epithelial barrier integrity (Paszti-Gere et al., 2015). However, a small study of COVID-19 patients and uninfected controls showed that measures of oxidative stress in sperm epithelial cells were not associated with levels of TMPRSS2 (Moghimi et al., 2021). Similarly, another experimental study showed that cigarette smoking extract (CSE) that is an established trigger of oxidative stress (Kau et al., 2016) had no effect on ACE2 and TMPRSS2 expression in endothelial cells (Youn et al., 2021). Overall, there is no solid evidence to support a role of increased redox stress in regulation of TMPRSS2.

Other than redox-dependent regulation of membrane receptors for coronaviruses, mito-ROS are also instigators of aberrant vacuole formation (Demers-Lamarche et al., 2016) by activation of adaptor-associated kinase 1 (AAK1), a regulator of endocytosis (Chen et al., 2006) that has been targeted therapeutically in SARS-CoV-2 infection with baricitinib (Stebbing et al., 2020). Mito-ROS can also induce alterations in membrane lipid rafts and lipid-based cellular signaling changing their properties (Morris et al., 2016) and these membrane changes may also impact viral entry of coronaviruses. Thus, redox mechanisms may regulate entry of coronaviruses in mammalian cells but these mechanisms need to be further studied specifically in airway epithelial cells and *in vivo*.

### Redox mechanisms that regulate cytoplasmic replication of coronavirus

Viral infections may alter the mitochondrial dynamics leading to excessive mito-ROS generation, mitochondrial biogenesis, and altered mitochondrial β-oxidation (Elesela and Lukacs, 2021). Mitochondria are targeted by coronavirus (Shi et al., 2014). Coronaviruses may directly induce production of mito-ROS in cells. Non-structured viral proteins, such as coronavirus 3a protein directly activate NLRP3 inflammasome in macrophages, which is mediated by increased mito-ROS level (Zhou et al., 2011; Chen et al., 2019). Finally, redox pathways also regulate cellular machinery that propagates replication of coronaviruses through multiple pathways.

First, Mito-ROS regulate the endoplasmic reticulum stress and the unfolded protein response (UPR) that contribute to replication of coronaviruses (de Wilde et al., 2011; Hyser and Estes, 2015; Kindrachuk et al., 2015; Zhang et al., 2016) and associated Ca^2+^ signaling systems. Second, mito-ROS induce the mitochondrial permeability transition pore (mPTP) that is a proviral factor for replication of coronaviruses. Indeed, by blocking the mPTP, cyclosporin A impacts coronavirus replication (Halestrap et al., 2004). Mitochondria-targeted antioxidants inhibit mPTP, mito-ROS (Halestrap et al., 2004), and ROS (Dikalova et al., 2010; Dikalov et al., 2014). Third, mito-ROS regulate mitophagy that regulates replication of coronaviruses. Protein misfolding mitochondrial depolarization and ROS activate mitophagy (Shirihai et al., 2015). Viral proteins like SARS-CoV ORF-9 (Shi et al., 2014) interact with mitophagic machinery such as LC3 and Beclin1 (Zhang et al., 2018). Therapeutic targeting of aberrant autophagy through Beclin1 reduces MERS infection (Gassen et al., 2019). Fifth, mito-ROS trigger MEK (Zhang et al., 2016), MNK1 (Wang and Zhang, 1999) and MAPK signaling pathways (Kulisz et al., 2002; Emerling et al., 2005; Trempolec et al., 2017) that propagate viral protein synthesis and SARS-Co-V replication (Mizutani et al., 2004; Kefaloyianni et al., 2006; Jamaluddin et al., 2009). Sixth, ROS regulate cytoplasmic interferon host antiviral responses during coronavirus infection. ROS promotes MHV replication by downregulating interferon host responses during MHV infection (Tao et al., 2020). Lastly, preclinical studies suggest that mito-ROS may contribute to viral reservoirs and replication of SARS-CoV-2 in macrophages, but this has not been clearly demonstrated *in vivo* (Codo et al., 2020). Thus, mito-ROS induce multiple proviral cytoplasmic pathways.

### Antioxidant mechanisms that regulate cytoplasmic replication of coronavirus

The primary transcription factor regulating the antioxidant response is the nuclear factor E2-related factor 2 (Nrf2), which regulates the Kelch-like ECH-associated protein 1 (Keap1)-Nrf2-antioxidant response elements (ARE) pathway (Khomich et al., 2018). Under normal circumstances, the Keap1-Nrf2-ARE pathway is activated by the oxidative stress resulting from ROS production. Nrf2, which is usually bound to Keap1 by ubiquitination or degraded by Keap1 in the absence of oxidative stress, is translocated to the nucleus when oxidative stress modifies the conformational structure of Keap1 and prevents it from binding Nrf2 (Komaravelli and Casola, 2014; Han et al., 2021). Mito-ROS activate Nrf2 through protein kinases, and induce production of antioxidant proteins and genes involved in mitochondrial quality control (Kasai et al., 2020). The activation of Nrf2 results in the upregulation of antioxidant gene expression as Nrf2 binds to antioxidant response element (ARE) sites, leading to the expression of key players of the antioxidant response, including heme oxygenase-1 (HO-1), NADPH quinone oxidoreductase 1 (NQO-1), superoxide dismutases (SOD), and glutathione derived molecules catalase, peroxiredoxins, and glutathione peroxidases which collectively attenuate oxidative stress (Khomich et al., 2018; Tu et al., 2019).

Several studies have found that respiratory viruses downregulate the expression of antioxidant genes by inhibiting Nrf2, preventing it from mobilizing to the nucleus and binding to ARE sites (Komaravelli and Casola, 2014). The Nrf2 pathway that mediates pathogenesis and tissue damage of several viral infections including HIV, RSV (Cho et al., 2009; Hosakote et al., 2009; Komaravelli et al., 2015), influenza (Kesic et al., 2011; Kosmider et al., 2012; Yamada et al., 2012; Simon et al., 2015), and SARS-CoV-2 (Olagnier et al., 2020). Induction of the Nrf2 pathway and key downstream mediators such as Heme oxygenase-1 (HO-1) triggers stress-inducible, anti-inflammatory, and antiviral responses present in most human cells (Espinoza et al., 2017). NRF2 has antiviral properties but, it remains unclear which genes mediate these effects and how they exert antiviral effect (Herengt et al., 2021).

Emerging evidence has increased our understanding of the role of Nrf2 activation in SARS-CoV-2 infection. *In vitro* experiments with Vero hTMPRSS2 cells, Calu-3 and primary human airway epithelial cell lines and using gene silencing of Keap1 and Nrf2 agonists 4-octyl-itaconate (4-OI) and dimethyl fumarate (DMF), it was shown that the Nrf2 pathway has a critical role in inhibiting SARS-CoV-2 replication, in addition to limiting the host inflammatory response. SARS-CoV-2 reduced *in vitro* basal levels of HO-1 and NQO-1 in lung cells. Notably, considering Nrf2’s known role in inhibiting anti-viral IFN responses, it was shown that the antiviral effect of Nrf2 is independent of interferon responses (Olagnier et al., 2020). Mechanistic preclinical studies showed that Nrf2 activation reduced SARS-CoV-2 replication by inducing the metabolite biliverdin, whereas SARS-CoV-2 altered the NRF2 axis through the cross-talk between the nonstructural viral protein NSP14 and the NAD-dependent deacetylase Sirtuin 1 (SIRT1; Olagnier et al., 2020; Zhang et al., 2022).

Experimental studies have also shown that downregulation of antioxidant genes by SARS-CoV-2 and SARS-CoV-1 is combined with an upregulation of oxidative stress genes like myeloperoxidase (MPO), calprotectin (S100A8 and S100A9), sulfiredoxin-1 (SRXN1), glutamate cysteine ligase modifier subunit (GCLM), sestrin2 (SESN2), and thioredoxin-1 (TXN; Saheb Sharif-Askari et al., 2021). The results of these studies have revealed key aspects of SARS-CoV-2 infection: such as downregulation of host’s antioxidant pathway as an important role in viral replication, and possible utility of activators of antioxidant pathways as specific therapeutic targets.

### Redox mechanisms that regulate replication of coronavirus through apoptotic pathways

Many viruses alter apoptosis or programmed cell death of the infected cell as a mechanism of increased production of virus progeny, cell killing and virus spread (Roulston et al., 1999). Apoptosis is the programmed cell death that involves the activation of proteases called caspases and a cascade of events that link apoptosis-initiating stimuli to final death of the cell. ROS (Pierce et al., 1991; Kasahara et al., 1997) and mitochondria play pivotal roles in induction of apoptosis under both physiologic and pathologic conditions. Increased mito-ROS induce apoptosis and cell death (Orrenius et al., 2007). Excessive ROS can activate pro-apoptotic Bcl-2 family proteins by increasing mitochondrial permeability to drive the mitochondrial membrane potential, release cytochrome c, mtDNA (Santos et al., 2003), and pro-apoptotic caspase-3 and-9. This leads to the activation of intrinsic or mitochondrial driven cell death by apoptosis (Green and Llambi, 2015). Coronaviruses impact apoptosis through several pathways. Notably, mitochondrial apoptosis is directly and uniquely induced by SARS-CoV (Pfefferle et al., 2011) triggering viral replication (Supinski et al., 2009; Maiti et al., 2017). SARS-CoV-2 infection also downregulates the Nrf2 pathway (Olagnier et al., 2020; Zhang et al., 2022) which has antiapoptotic cellular effect (Niture and Jaiswal, 2012; Khan et al., 2018). Thus, coronaviruses induce apoptosis through multiple pathways, either directly (Pfefferle et al., 2011), or indirectly by inducing production of mito-ROS and downregulating antiapoptotic pathways such as Nrf2 and the virus-induced alteration of mitochondrial apoptosis contributes to increased replication of coronaviruses (Supinski et al., 2009; Pfefferle et al., 2011; Maiti et al., 2017).

### Redox mechanisms that regulate replication of coronavirus through the complement system

The complement system is a major host defense mechanism against viral replication. Several viruses hijack the complement system for cellular entry and spread (Agrawal et al., 2017). The role of the complement system in the pathogenesis of coronavirus infections is complex and contradictory (Santiesteban-Lores et al., 2021). During SARS-CoV-2 infection, the complement system is a host defense mechanism against viral replication in asymptomatic or mild cases (Santiesteban-Lores et al., 2021). However, complement activation has also potent proinflammatory effect and can increase local and systemic damage in severe COVID-19 (Santiesteban-Lores et al., 2021). As outlined above, coronavirus induce production of mito-ROS during infection. Mito-ROS induce the “complement–metabolism–inflammasome axis”(Arbore and Kemper, 2016). MERS-CoV can also directly induce the complement system (Chen et al., 2010). Collectively, limited evidence suggests that complement activation through redox pathways may have a more important role in cell and tissue damage in severe coronavirus infections rather than a major regulatory role in replication of coronaviruses.

### Redox mechanisms that regulate replication of coronavirus through mitophagy

Mitophagy, the cellular process that clears excess or damaged mitochondria, has a key role in function of mitochondria and mammalian cells and regulates severeal physiological and pathological processes, including apoptosis, immunity and inflammation. Emerging evidence suggests that several viruses hijack mitophagy to enable viral replication and escape host immune responses (Li et al., 2022). SARS-CoV can encode open reading frame-9b (ORF-9b), which is localized in mitochondria and induces mitochondrial elongation which further triggers mitophagy and coronavirus replication (Shi et al., 2014). Preclinical studies have shown that SARS-CoV-2 directly causes mitochondrial dysfunction and mitophagy impairment (Shang et al., 2021). Notably, defects in autophagy and mitophagy processes may regulate host response to coronavirus infection (Pacheco et al., 2021). Coronaviruses also induce production of mito-ROS that have an established complex crosstalk with mitophagy (Schofield and Schafer, 2021). Overall, further evidence is needed to clearly link the role of aberrant redox pathways and mitophagy in the regulation of replication of coronaviruses.

## Redox pathways that regulate inflammation during infection with coronaviruses

Several redox mechanisms regulate inflammation during infection with coronaviruses (Figure 2; Table 2; Shono et al., 1996; Wesselborg et al., 1997; Chua et al., 1998; Canty et al., 1999; Tenjinbaru et al., 1999; Wang and Zhang, 1999; Cooke and Davidge, 2002; Pearlstein et al., 2002; Takada et al., 2003; Mizutani et al., 2004; Desouki et al., 2005; Mukherjee et al., 2005; Kefaloyianni et al., 2006; Xie and Shaikh, 2006; Schrader et al., 2007; Doughan et al., 2008; Nanduri et al., 2008; Cho et al., 2009; Hosakote et al., 2009; Jamaluddin et al., 2009; Martinon et al., 2009; Wosniak et al., 2009; Dikalova et al., 2010; Bulua et al., 2011; Kesic et al., 2011; Kosmider et al., 2012; Yamada et al., 2012; Lee et al., 2013; Nakajima and Kitamura, 2013; Nguyen Dinh Cat et al., 2013; Komaravelli and Casola, 2014; Zinovkin et al., 2014; Komaravelli et al., 2015; Simon et al., 2015; Sun et al., 2016; Zhang et al., 2016; Daiber et al., 2017; Espinoza et al., 2017; Khomich et al., 2018; Tu et al., 2019; Valle et al., 2019; Connors and Levy, 2020; Mahmud-Al-Rafat et al., 2020; Olagnier et al., 2020; Herengt et al., 2021; Saheb Sharif-Askari et al., 2021; Toro et al., 2022).

**Table 2:** Redox mechanisms that regulate cell and tissue damage during infection with coronaviruses.

Mediators	Effect on redox balance	References
Redox NF-kB	Context-dependent since ROS can in theory ↑ or ↓ NF-kB (e.g., phase of responses, pattern of stimulation, cell types of kB, etc).	Nakajima and Kitamura (2013)
	Overall evidence supports that ROS ↑ NF-kB during acute infection	
	Drive cytokine storm, triggering lung damage during viral infection	
ROS	↑ NF-kB	Pearlstein et al. (2002), Mukherjee et al. (2005), Zinovkin et al. (2014)
	↑ TNF-induced IL-6 expression.	
	↑ TNF-dependent ↑ expression of the adhesion molecules and ↑ endothelial permeability.	
	↑ apoptosis and cell/tissue damage	
	↑ end organ disease (brain, lung, cardiometabolic damage) in Long COVID	
Mito-ROS	↑ NF-kB	Shono et al. (1996), Wesselborg et al. (1997), Chua et al. (1998), Canty et al. (1999), Tenjinbaru et al. (1999), Wang and Zhang (1999), Cooke and Davidge (2002), Mizutani et al. (2004), Desouki et al. (2005), Kefaloyianni et al. (2006), Xie and Shaikh (2006), Doughan et al. (2008), Jamaluddin et al. (2009), Martinon et al. (2009), Wosniak et al. (2009), Bulua et al. (2011), Lee et al. (2013), Sun et al. (2016), Zhang et al. (2016), Daiber et al. (2017), Saheb Sharif-Askari et al. (2021)
	↑ complement-metabolism-inflammasome axis	
	↑ Indirectly inflammatory caspases 1, 12, cytokines IL-1B, IL-18 through NLRP3 inflammasome	
	↑ activation of MAPK, MEK, MNK1 pathways →↑ production of IL-6 and TNF-a	
	↑ induce release of IL-1B, IL-6 and lung injury under viral infection	
	↑ Mito-ROS regulates NOX and impacts survival rates of mice with post-viral pneumonia	
	Regulate Ca^2+^ signaling systems that may impact inflammatory host responses	
	↑ Nrf2 pathway	
	Regulate ER stress and unfolded protein response that may impact inflammatory host responses	
	Regulate interferon host responses	
	↑ apoptosis and cell/tissue damage	
	Regulate mitophagy/autophagy and cell/tissue damage	
Keap1-Nrf2-ARE pathway	↑ anti-viral responses	Cho et al. (2009), Hosakote et al. (2009), Kesic et al. (2011), Yamada et al. (2012), Kosmider et al. (2012), Komaravelli and Casola (2014), Komaravelli et al. (2015), Simon et al. (2015), Espinoza et al. (2017), Khomich et al. (2018), Tu et al. (2019), Olagnier et al. (2020), Herengt et al. (2021), Toro et al. (2022)
	↑ anti-inflammatory responses	
	Remove toxic heme	
	Protect against oxidative injury	
	↑ anti-apoptotic responses	
	Regulates angiogenesis	
	Regulates autoimmunity	
	Regulates vascular injury	
	Mediates pathogenesis and tissue damage of many viral infections, including HIV, RSV, Influenza, SARS-CoV-2	
	↓ apoptosis that regulates cell death and tissue damage	
Ang II	↑ ROS levels through NADPH oxidase, →↑ cytokines (e.g., IL-6, IL-8, TNF-a) through NF-kB upregulation →↑ pro-inflammatory response	Nguyen Dinh Cat et al. (2013), Mahmud-Al-Rafat et al. (2020)
Type I IFNs	Coronaviruses and ROS downregulate interferon host responses that impact a cascade of signaling events that may drive tissue damage	Dikalova et al. (2010)
Cytokines (bidirectional link with redox stress)	Cytokines (e.g., IL-1, IL-6, TNFa) activate macrophages, neutrophils, endothelial cells through NOX, disrupting redox balance of the cell	Takada et al. (2003), Schrader et al. (2007), Nanduri et al. (2008), Valle et al. (2019), Connors and Levy (2020)
	IL-6 directly induces mito-ROS production and NOX in endothelial cells	

Abbreviations: ACE2, Angiotensin-converting enzyme 2; AngII, Angiotensin II; ARE, antioxidant response element; COVID, COrona Virus Disease; Ca^2+^, Calcium (II) ion; ER, endoplasmic reticulum; HIV, human immunodeficiency virus; HO-1, Heme oxygenase 1; IFNs, Interferons; IL, interleukin; Keap1, Kelch-like ECH-associated protein 1; MAPK, mitogen-activated protein kinase; MEK, Mitogen-activated protein kinase; Mito-ROS, Mitochondrial reactive oxygen species; Mnk1, mitogen-activated protein kinase (MAPK) interacting protein kinase 1; NF-κB, Nuclear factor kappa B; NOX, nicotinamide adenine dinucleotide phosphate (NADPH) oxidase; Nrf2, nuclear factor erythroid 2–related factor 2; NQo-1, NAD(P)H quinone oxidoreductase; ROS, reactive oxygen species; RSV, Respiratory Syncytial Virus; SARS-CoV-2, severe acute respiratory syndrome coronavirus 2; TNF, Tumor necrosis factor; UPR, unfolded protein response.

### NF-κB pathway

Nuclear factor-κB (NF-κB) is a redox-sensitive transcription factor that is regulated by ROS through the classical IkB kinase (IKK)-dependent canonical pathway (Liu et al., 2017) and coordinates innate and adaptive immunity, inflammation, and apoptosis (Piette et al., 1997). The redox regulation of the NF-κB pathway has been reviewed elsewhere and varies between different mammalian cells and in the setting of cancer (Gloire et al., 2006). Although it is established that cytokines and lipopolysaccharides induce proinflammatory activation of NF-κB (Schreck and Baeuerle, 1991), ROS may also reduce NF-κB activity (Nakajima and Kitamura, 2013). Oxidative stress in the early phase may induce activation of NF-κB in epithelial cells (Wesselborg et al., 1997; Tenjinbaru et al., 1999; Thevenod et al., 2000) and endothelial cells (Shono et al., 1996; Chua et al., 1998; Canty et al., 1999; Cooke and Davidge, 2002) which are targets of coronaviruses. Redox stress in epithelial cells in the late phase may also inhibit basal and inducible activation of NF-κB (Xie and Shaikh, 2006; Yang et al., 2007; Nakajima and Kitamura, 2013). The regulation of NF-κB by ROS is dependent not only on the phase of responses and the pattern of stimulation, but also depends on specific cell types (Nakajima and Kitamura, 2013). However, most of the evidence regarding redox regulation of the NF-κB pathway is not based on airway epithelial cells, the main target of SARS-CoV-2, and heterogeneous redox stimuli have been utilized in several experimental studies, often in supraphysiological concentrations. Thus, it is not well defined how ROS regulate activity of NF-κB in a bidirectional fashion in airway epithelial cells (Nakajima and Kitamura, 2013).

Overall, cumulative evidence suggests that there is context-dependent regulation of NF-κB by ROS (Nakajima and Kitamura, 2013). Preclinical studies have shown that ROS trigger NF-κB activation in airway epithelial cells (Jany et al., 1995; Ito et al., 2004). In contrast, inhibition of cytokine-triggered NF-κB activation under pre-exposure to ROS has been described in distal airway alveolar epithelial cells (Korn et al., 2001; Reynaert et al., 2006). The oxidative stress– unfolded protein response (UPR) pathway and redox ER responses play a key role in the bidirectional control of NF-κB (Nakajima and Kitamura, 2013). Thus, the opposite, bidirectional effects of redox stimuli on NF-κB seem to depend on the phase of response, the context, the type of cells and the specific redox stimuli. Overall, this bidirectional crosstalk is not well characterized specifically in coronavirus infections.

Viruses may hijack cellular signaling pathways and transcription factors and control them to their own advantage. In particular, the NF-κB pathway appears to be an attractive target for common human viral pathogens (Santoro et al., 2003). Distinct viral proteins encoded by viruses such as HCV, rotavirus, EBV, HBV, HTLV-1, and HIV-1 activate NF-κB by interacting with cellular signaling pathways including calcium-or redox-regulated signals or through ER stress mechanisms. Accumulation of viral dsRNA activates PKR, which in turn stimulates IKK. However, most of the evidence regarding virus-induced regulation of the NF-κB pathway is based on chronic viral infections or infections with DNA viruses (Santoro et al., 2003). There is limited evidence regarding the direct impact of coronaviruses on this pathway.

Evidence has suggested that proteins of SARS-CoV-2 can directly or indirectly impact NF-kB activation. *In vitro* studies showed that the spike protein of SARS-CoV induces a strong cytokine response through the NF-kB pathway (Dosch et al., 2009). It was also shown that SARS-CoV nucleocapsid protein activated NF-kB in Vero E6 cells in a dose dependent manner (Liao et al., 2005). ORF7a protein of SARS-CoV-2 mediates activation of NF-kB and induced proinflammatory expression of cytokines (Su et al., 2021). Similarly, Nsp5 in SARS-CoV-2 activated NF-kB pathway through upregulation of SUMOylation of mitochondrial antiviral-signaling proteins (Li et al., 2021b). Notably, studies show that the NF-κB signal pathway is a central pathway involved in induction of pro-inflammatory cytokines and chemokines in respiratory virus infection, including SARS-CoV-2-triggered COVID-19 (Kircheis et al., 2020; Hariharan et al., 2021; Kandasamy, 2021). Thus, the pharmacological inactivation of the NF-κB signaling pathway can represent a potential therapeutic target to treat severe COVID-19 (Kircheis et al., 2020; Hariharan et al., 2021; Kandasamy, 2021).

### Mito-ROS pathways

As outlined above, coronavirus induce production of mito-ROS during infection. Mito-ROS have been shown to inhibit interferons and induce aberrant alterations of lipids, membranes, proteins and ultimately tissue damage. Mito-ROS induce inflammasome activation (Dashdorj et al., 2013; Han et al., 2018) and the “complement–metabolism–inflammasome axis”(Arbore and Kemper, 2016), Mito-ROS indirectly regulate inflammatory caspases 1 and 12, as well as the cytokines IL-1β and IL-18 in macrophages through the NLRP3 inflammasome (Martinon et al., 2009). Mito-ROS induce NFκB (Imai et al., 2008) which drives a cytokine storm, triggering lung damage during viral infection. Mito-ROS also induce activate MAPK pathways and promote production of IL-6 and TNF-α (Wang and Zhang, 1999; Mizutani et al., 2004; Kefaloyianni et al., 2006; Jamaluddin et al., 2009; Bulua et al., 2011; Zhang et al., 2016). Mito-ROS directly induce release of IL-1β (Dashdorj et al., 2013; Han et al., 2018), IL-6 (Lowes et al., 2008, 2013; Bulua et al., 2011; Li et al., 2019). Consistent with this evidence it has been shown that Mito-ROS induce inflammatory response and lung injury in mouse models of viral infections (Hu et al., 2019a; Hu et al., 2019b). Thus, mito-ROS may regulate redox cytoplasmic proinflammatory responses in respiratory viral infections.

### Nf2 pathways

Heme oxygenase 1 (HO-1), a downstream protein of the Nrf2 pathway, contributes to anti-inflammatory and antiviral responses, removes toxic heme, protects against oxidative injury and also regulates apoptosis, inflammation and angiogenesis (Espinoza et al., 2017). While the exact mechanism by which SARS-CoV-2 affects HO-1 and, conversely, how HO-1 exerts its antiviral effects against SARS-CoV-2 is still being studied, there is an established association between HO-1 and a reduction of tissue damage through its anti-inflammatory and antioxidative functions throughout the body (Toro et al., 2022). This makes HO-1 an important target for developing novel COVID-19 therapeutics.

### Angiotensin II and NOX

During SAS-CoV-2 infection, the reduction of ACE2 on the cell surface leads to increase of Ang II and NOX (Nguyen Dinh Cat et al., 2013). Bidirectional crosstalk between mitochondria and NOX, markedly affects redox responses to angiotensin II, the ligand of ACE2 that is upregulated in viral infections (Doughan et al., 2008; Wosniak et al., 2009; Lee et al., 2013; Daiber et al., 2017). Indeed, therapeutic targeting of NOX, triggered by mito-ROS (Desouki et al., 2005), increased the survival of mice with post-influenza pneumonia (Sun et al., 2016). Thus, as a result of increased NOX, NF-κβ activation there is activation of the pro-inflammatory response and release of cytokines like IL-6, IL-8, and TNFα (Mahmud-Al-Rafat et al., 2020). Pro-inflammatory cytokines like IL-1, IL-6, and TNFα activate macrophages, neutrophils, and endothelial cells through NADPH oxidase, resulting in a greater production of superoxide and H2O2 (Takada et al., 2003; Nanduri et al., 2008; Connors and Levy, 2020).

### The complement system

As outlined above, coronavirus induce production of mito-ROS which trigger the “complement–metabolism–inflammasome axis”(Arbore and Kemper, 2016). MERS-CoV can also directly induce the complement system (Chen et al., 2010). The complement activation has also potent proinflammatory effect and can increase local and systemic damage in severe COVID-19 (Santiesteban-Lores et al., 2021). Preclinical *in vitro* studies have shown controversial data regarding the role of the complement system in binding coronaviruses (Santiesteban-Lores et al., 2021). Experimental studies with animals have shown that complement activation induces a systemic pro-inflammatory response during experimental infection with SARS-CoV and MERS that drives disease progression (Gralinski et al., 2018; Jiang et al., 2018). Small human cohorts also show that complement activation is associated with disease progression of SARS (Wang et al., 2005). Collectively, limited and often controversial evidence suggests that complement activation through redox pathways may have an important role in cell and tissue damage in severe coronavirus infections.

### Other proinflammatory mechanisms in coronavirus infections

Other than activation of proinflammatory NF-kB, mito-ROS and NOX pathways and downregulation of anti-inflammatory ACE2 and Nrf2 pathways, different coronaviruses may also directly induce other proinflammatory effects. MERS-CoV can induce the complement system and increase inflammatory response, pyroptosis and eventually lung tissue damage (Chen et al., 2010). MERS-CoV infected macrophages increase pro-inflammatory cytokines and chemokines (Pruijssers and Denison, 2019). SARS-CoV and MERS-CoV may also attenuate levels of endogenous Type I IFNs that are immunomodulatory (Dikalova et al., 2010). Finally, mouse hepatitis virus (MHV) directly upregulated interleukin signaling such as IL-27 during acute encephalomyelitis.

## Redox pathways that regulate apoptosis during infection with coronaviruses

As described above, coronaviruses induce apoptosis through multiple pathways, either directly (Pfefferle et al., 2011), or indirectly by inducing production of mito-ROS and downregulating antiapoptotic pathways such as Nrf2. Excessive ROS generation can lead to loss of mitochondrial function and apoptosis of lung epithelial cells (Sun et al., 2013). Increased mito-ROS also directly contribute to acute injury in lung tissue in mouse models of viral infections (Hu et al., 2019a,b). Indeed, increased apoptosis of epithelial cells is associated with lung injury in COVID-19 (Hussman, 2020). Studies have also shown that CD4 and CD8 T cells in patients with COVID-19 are more likely to get affected by apoptosis (Nieto-Torres et al., 2015). Thus, increased apoptosis during coronavirus infection contributes to increased tissue damage and pathogenesis of coronavirus infections.

## Redox pathways that regulate mitophagy during infection with coronaviruses

As outlined above, coronaviruses induce production of mito-ROS that have an established complex crosstalk with mitophagy (Schofield and Schafer, 2021). Mitochondrial ROS and damage-associated molecular patterns (DAMPs) activate inflammasomes to induce inflammatory responses and tissue injury. Emerging evidence suggests that mitophagy protects against the hyperinflammation induced by ROS and DAMPs and regulates inflammatory responses in several diseases (Zhao et al., 2015). Thus, by inducing production of mito-ROS, mitochondrial dysfunction and mitophagy impairment, SARS-CoV-2 may contribute to inflammation and tissue damage (Shang et al., 2021).

## Redox pathways that regulate other instigators of tissue damage during infection with coronaviruses

Other than regulation of viral replication, inflammation and apoptosis, redox pathways may also contribute to regulation of other pathways that contribute to tissue damage such as autoimmunity and vascular dysfunction. Oxidative stress plays a central in autoimmune diseases (Ramani et al., 2020). Specifically, the antioxidant pathway Nrf2 has also a key role in regulation of autoimmunity (Freeborn and Rockwell, 2021). Given the possible role of autoimmunity in pathogenesis of COVID-19 and Long COVID, further understanding of the contribution of dysregulation redox pathways in development of autoimmunity during coronavirus infections is needed (Liu et al., 2021; Saad et al., 2021).

ROS induce levels of the adhesion molecules and increase permeability in endothelial cells (Mukherjee et al., 2005; Zinovkin et al., 2014). ROS also contribute to TNF-induced IL-6 expression and NF-κB activation (Pearlstein et al., 2002). Notably, IL-6 directly induces mito-ROS production and NOX in endothelial cells (Schrader et al., 2007; Valle et al., 2019) and impact NO bioavailability and endothelial function (Saura et al., 2006). SARS-CoV-2 S-protein binds to ACE2 and subsequently triggers reduction in ACE2 levels that cleaves ATII. High ATII level further leads to oxidative stress and endothelial dysfunction (Chernyak et al., 2020) and induces ROS production *via* NOX in endothelial cells. Thus, increased redox stress induced by SARS-CoV-2 may impact not only vascular permeability and vasodilation but also vascular inflammation.

## Oxidative stress and end organ damage during infection with coronaviruses

**Figure 3:**
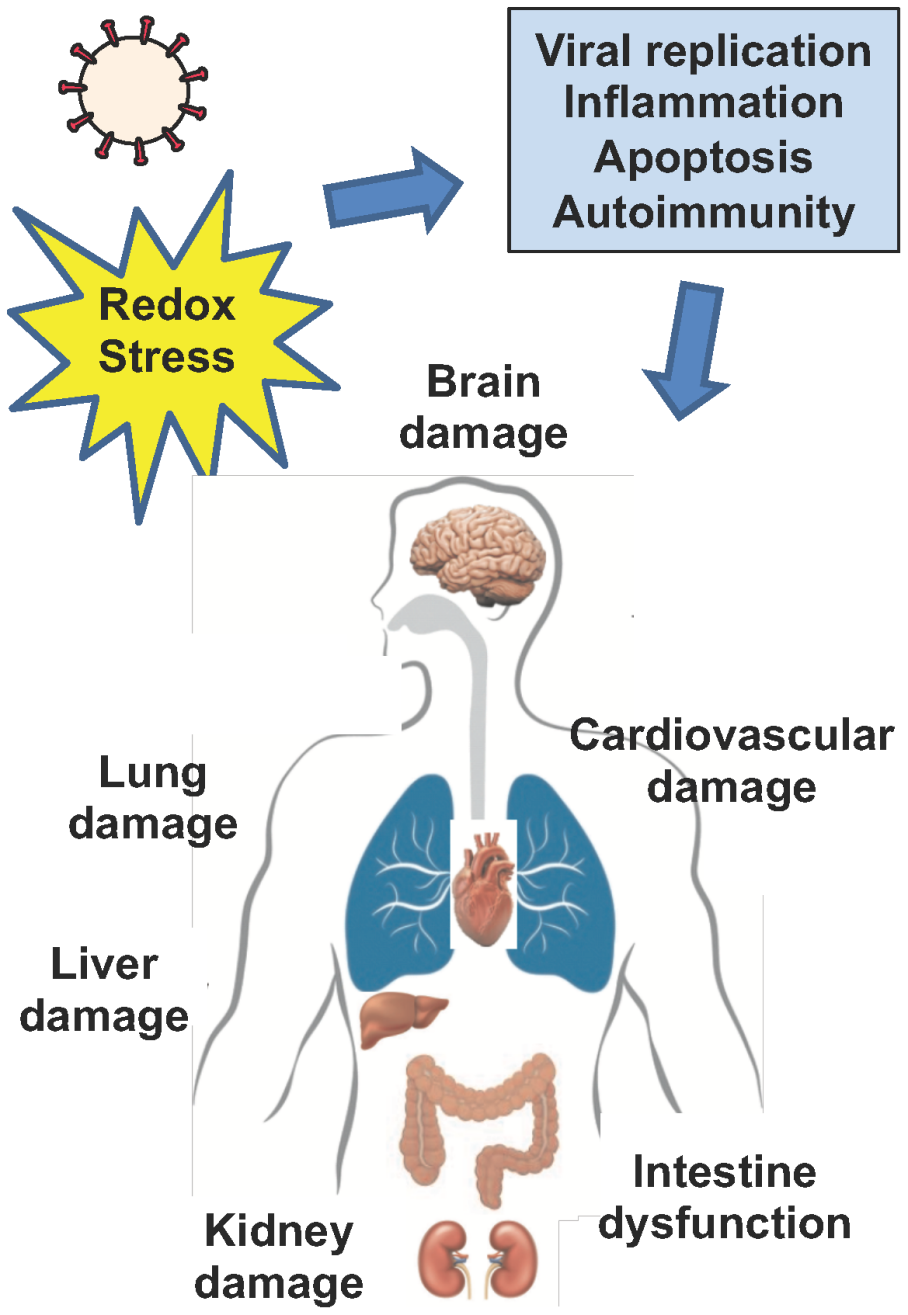
Viral infection and end organ damage. Increased redox stress drives viral replication, inflammation, apoptosis, vascular dysfunction and autoimmunity, in both acute infection with coronaviruses and in the setting of Post-Acute Sequelae of SARS-CoV-2 (PASC or Long COVID). Collectively, redox mediated pathways that drive viral replication, inflammation, apoptosis, autoimmunity, and vascular dysfunction contribute to cell and tissue damage that drives end organ disease in coronavirus infection. Cells enriched in mitochondria such as neurons, endothelial and epithelial cells may be particularly susceptible to increased redox stress driven by coronaviruses. Ultimately, increased redox stress during acute infection with coronaviruses and in the setting of PASC can directly or indirectly drive end organ disease such as brain, lung, liver, kidney and cardiovascular damage and induce intestinal dysfunction.

All coronaviruses have the potential to induce tissue damage and end organ disease through viral replication, increased inflammation and apoptosis, induction of ROS and reduction of cytoprotective pathways such as the Nrf2 and HO-1 pathways. Increased redox stress is known instigator of lung dysfunction (Kellner et al., 2017), cardiovascular disease (Dubois-Deruy et al., 2020), central nervous system dysfunction such as neurodegeneration and neuropsychiatric disease (Reiter, 1998; Patel, 2016; Salim, 2017) and the metabolic syndrome (Ando and Fujita, 2009; Roberts and Sindhu, 2009; Carrier, 2017) which are all manifestations of both acute severe COVID-19 and post-acute sequelae of SARS-CoV-2 infection (often called Long COVID syndrome; Figure 3; Nalbandian et al., 2021). Coronaviruses differ in their potential to induce end organ damage (Table 3; Bonavia et al., 1997; De Albuquerque et al., 2006; de Wilde et al., 2013; Josset et al., 2013; Zhao et al., 2015; Li et al., 2016; Agostini et al., 2018; Coperchini et al., 2020; Huang et al., 2020; Petersen et al., 2020; Wang et al., 2020; Yi et al., 2020; Caldera-Crespo et al., 2021; Paidas et al., 2021; Tian et al., 2021; Jansen et al., 2022). Among the various human coronaviruses, end organ damage is observed in MERS, SARS-CoV-1, and SARS-CoV-2. These coronaviruses demonstrate a more severe pathology than HCoV-229E, HCoV-OC43, HCoV-NL63, and HCoV-HKU1 in terms of their fatality and systemic effects on multiple organ systems. Multiple animal models have been used to uncover the ways coronaviruses lead to the end organ damage that presents in patient autopsies. The MHV mice model is the most studied model among the coronaviruses, and it has served as a useful proxy in understanding SARS-CoV-2 (Paidas et al., 2022); MHV is known to enteric and respiratory disease, hepatitis, encephalitis, and chronic demyelination and is useful in studying infection of the liver and brain (Weiss and Navas-Martin, 2005). Despite MHV-1 utilizing a different receptor than either MERS or the SARS coronaviruses (carcinoembryonic antigen-related cell adhesion molecule 1 instead of dipeptidylpeptidase 4 and angiotensin-converting enzyme 2), end organ damage in the MHV-1 model has been acclaimed as an appropriate model for MERS, SARS-CoV-1, and SARS-CoV-2 (De Albuquerque et al., 2006; Agostini et al., 2018; Caldera-Crespo et al., 2021; Paidas et al., 2021; Tian et al., 2021). Herein, we briefly summarize redox pathways that regulate damage of the lung and the brain, the two main target organs for end organ disease in acute COVID-19 and Long COVID.

**Table 3:** Comparison of coronaviruses with regards to impact on end organ disease.

Differences SARS-CoV-2 with other coronaviruses	Similarities between SARS-CoV-2 with other coronaviruses
↑ Transmissibility and ↑ affinity to the ACE2 receptor compared to other coronaviruses (Coperchini et al., 2020).	↑ cytokine storm, severe pneumonia, septic shock and multiorgan damage similarly to SARS and MERS
↑ Viral replication compared to SARS (Huang et al., 2020).	Infects the airways
↑ Cytokine storm similarly to SARS	Impacts the brain similarly to MHV (De Albuquerque et al., 2006; Agostini et al., 2018; Caldera-Crespo et al., 2021; Paidas et al., 2021; Tian et al., 2021), HCoV-OC43 and HCoV-229E (Bonavia et al., 1997).
↑ Cytokine TH1 pro-inflammatory cytokines compared to SARS (Huang et al., 2020).	Infects the liver (Wang et al., 2020) similarly to MHV (De Albuquerque et al., 2006; Agostini et al., 2018; Caldera-Crespo et al., 2021; Paidas et al., 2021; Tian et al., 2021) and MERS (Zhao et al., 2015).
↓ Interferon response compared to SARS and MERS (Li et al., 2016)	Impacts the heart similarly to MHV (De Albuquerque et al., 2006; Agostini et al., 2018; Caldera-Crespo et al., 2021; Paidas et al., 2021; Tian et al., 2021).
Unlike MERS requires a TH17 type response (Yi et al., 2020)	Impacts the kidney (Jansen et al., 2022) similarly to MHV (De Albuquerque et al., 2006; Agostini et al., 2018; Caldera-Crespo et al., 2021; Paidas et al., 2021; Tian et al., 2021).
↓ Severe symptoms compared to SARS/MERS (Petersen et al., 2020).	↑ vascular injury and thrombosis (Siddiqi et al., 2021) similarly to MHV (De Albuquerque et al., 2006; Agostini et al., 2018; Caldera-Crespo et al., 2021; Paidas et al., 2021; Tian et al., 2021).
MERS is more cytopathic and causes greater immune system dysregulation compared to SARS-CoV-2 (de Wilde et al., 2013; Josset et al., 2013)	

Abbreviations: ACE2, Angiotensin-converting enzyme 2; HCoV-229E, Human coronavirus 229E; HCoV-OC43; Human coronavirus OC43; HCoV-NL63; HKU-1, HCoV-HKU1 = human coronavirus HKU1; MERS-CoV, Middle East respiratory syndrome coronavirusl MHV, mouse hepatitis virus; SARS-CoV, Severe acute respiratory syndrome coronavirus; SARS-CoV-2, Severe acute respiratory syndrome coronavirus 2; TH1, Type 1 T helper

### Lung damage

The excessive generation of oxygen radicals under pathological conditions such as acute lung injury (ALI) and its most severe form acute respiratory distress syndrome (ARDS) leads to increased endothelial permeability. Increased redox stress leads to increased permeability of lung blood vessels, increased infiltration of immune cells and increased accumulation of fluids in the alveolar system (Kellner et al., 2017). Mitochondria, NADPH oxidase (NOX), xanthine oxidase (Shasby et al., 1985; Barnard and Matalon, 1992), and eNOS are the major contributors of ROS in cells of vasculature during active metabolism that also contribute to the pathogenesis of ALI (Gross et al., 2015). Imbalance of antioxidant enzymes such as superoxide dismutase (SOD; Ndengele et al., 2005; Cai et al., 2014), catalase (Flick et al., 1988; Kozower et al., 2003) and glutathione peroxidase (GPx; Aggarwal et al., 2012; Kim et al., 2012a) and Nrf2 (Zhu et al., 2013; Peng et al., 2016) also contribute to pathogenesis of ALI and ARDS. Similarly, to MERS and SARS, severe SARS-CoV-2 infection presents with high levels of pro-inflammatory cytokines like IL-6, and can lead to ARDS, which is associated with acute renal injury, acute respiratory injury, and septic shock (Chen et al., 2020). COVID-19-related ARDS has a high prevalence and is different to ARDS due to other etiologies (Park et al., 2009).

SARS-CoV-2 directly impacts several of established instigators that contribute to pathogenesis of ALI/ARDS including mitochondrial function (Srinivasan et al., 2021), NOX (Damiano et al., 2020; Violi et al., 2020; de Oliveira and Nunes, 2021), xanthine oxidase (Pratomo et al., 2021; Al-Kuraishy et al., 2022), eNOS (Guimaraes et al., 2021), glutathione peroxidase (Labarrere and Kassab, 2022) and Nrf2 (Olagnier et al., 2020; Zhang et al., 2022). To date, there is no treatment for ARDS in COVID-19 disease (Jafari-Oori et al., 2021).

### Brain damage

The brain is highly susceptible to oxidative stress due to enrichment for lipids, mitochondria, calcium, glutamate and increased redox stimuli (Cobley et al., 2018). Brain damage induced by oxidative stress may negatively impact normal functions of central nervous system and may contribute to the pathogenesis of neurodegenerative disorders such as Alzheimer and Parkinson disease and in the pathogenesis of neuropsychiatric disorders, including anxiety and depression (Salim, 2017). For these, increased oxidative stress through mitochondrial dysfunction, increased inflammation and energy imbalance has also been hypothesized to contribute to pathogenesis of neurocognitive dysfunction in Long COVID (Paul et al., 2021; Jarrott et al., 2022).

## Antioxidant therapies in coronavirus infections

Multiple trials underway have tested antioxidants as therapeutic agents in COVID-19.^1^ Several therapies targeting redox imbalance already have been used for the treatment of COVID-19 including inhaled NO (Lotz et al., 2021), ubiquinol (Fukuda et al., 2016), combination of NADH and CoQ10 (Castro-Marrero et al., 2015), N-acetyl cysteine, mitochondria-targeted antioxidant MitoQ (Codo et al., 2020; Petcherski et al., 2022) and Nrf2 agonists (Zinovkin and Grebenchikov, 2020). Other potential antioxidant treatments that have been considered include, ubiquinol, nicotinamide, glutathione (and glutathione donors), cysteamine, sulforaphane, melatonin vitamin C, vitamin D, vitamin E, melatonin plus pentoxifylline and selenium. However, most of the proposed antioxidant treatments have either not been directly tested in humans in the setting of randomized control clinical trials or due to several methodological issues of heterogeneous studies, the data were inconclusive (Table 4). Many ongoing clinical trials regarding the use of antioxidants in treatment of COVID-19 have not been published. Notably, oral antioxidants have not produced dramatic improvements in conditions associated with redox imbalance (Barcelos et al., 2020). No single antioxidant can scavenge all the various ROS and reactive nitrogen species (RNS). Further validation with animal models and clinical trials are necessary to reveal therapeutic potential of combination therapies of antivirals, antioxidant and anti-inflammatory treatments.

**Table 4:** Antioxidant treatments that have been tested in humans for treatment of coronavirus infections.

Mediators	Effect	References
Inhaled NO	↑ oxygenation in severe COVID-19, no effect on mortality	Lotz et al. (2021), Prakash et al. (2021)
Ubiquinol (CoQ10)	Does not ↓ the number or severity of PASC-related symptoms when compared to placebo	Hargreaves and Mantle (2021), Hansen et al. (2022)
N-acetyl cysteine	Oral high dose of N-acetyl cysteine may ↓ morbidity in severe COVID-19 in observational studies; many ongoing clinical trials with unpublished data	Wong et al. (2021), Izquierdo et al. (2022)
Glutathione	↓ reduces dyspnea in COVID-19 in a case series	Horowitz et al. (2020)
Melatonin	May improve clinical outcomes in patients with COVID-19 based on RCTs	Lan et al. (2022)
Vitamin C	Controversial data may have some benefit in morbidity in COVID-19 based on clinical trials	Olczak-Pruc et al. (2022)
Vitamins	Controversial data overall weak/negative; supplementation with vitamins A, B, C, D, and E could improve the inflammatory response and decrease the severity of disease in ICU-admitted patients with COVID-19	Beigmohammadi et al. (2021)
Zinc	Overall limited data/no major effect on morbidity in COVID-19, many ongoing clinical trials with unpublished data	Perera et al. (2020), Balboni et al. (2022)
Selenium	Overall limited data/no major effect on morbidity in COVID-19, many ongoing clinical trials with unpublished data	Alshammari et al. (2022), Balboni et al. (2022)
Pentoxifylline	May reduce lung inflammation, ongoing clinical trials with unpublished data	Feret et al. (2021)

Abbreviations: RCT, Randomized control clinical trial; PASC, Post Acute Sequalae of SARS-CoV-2 infection.

## Conclusion

There is limited understanding how different coronaviruses including SARS-CoV-2, manipulate cellular redox machinery to drive viral replication and associated host cell responses including inflammation, apoptosis and associated end organ disease. The crosstalk between NOX and ACE2 as well mito-ROS may impact viral entry of coronaviruses while mito-ROS may also induce multiple proviral cytoplasmic pathways. Experimental studies have also shown that coronaviruses induce downregulation of antioxidant genes such as Nrf2 in combination with an upregulation of oxidative stress genes like myeloperoxidase that may contribute to both increased viral replication and inflammation. Coronaviruses may induce several redox sensitive proinflammatory pathways such NF-kB, mito-ROS and NOX pathways and downregulate anti-inflammatory ACE2 and Nrf2 pathways. Coronaviruses may further trigger cell damage through activation of redox sensitive pyroptosis and apoptosis. Finally, other than regulation of viral replication, inflammation and apoptosis, redox pathways may also contribute to regulation of other pathways that contribute to tissue damage such as autoimmunity and vascular dysfunction. Thus, coronaviruses have the potential to induce tissue damage and end organ disease through viral replication, increased inflammation and apoptosis, induction of ROS and reduction of cytoprotective pathways such as the Nrf2 and HO-1 pathways. Coronaviruses differ in their potential to induce end organ damage. Among the various human coronaviruses, end organ damage is observed in MERS, SARS-CoV-1, and SARS-CoV-2. Increased redox stress is known instigator of lung dysfunction (Kellner et al., 2017), cardiovascular disease (Dubois-Deruy et al., 2020), central nervous system dysfunction such as neurodegeneration and neuropsychiatric disease (Reiter, 1998; Patel, 2016; Salim, 2017) and the metabolic syndrome (Ando and Fujita, 2009; Roberts and Sindhu, 2009; Carrier, 2017) which are all manifestations of both acute severe COVID-19 and Long COVID syndrome (Nalbandian et al., 2021). Given the complexity of the pathogenesis of coronavirus infections and that oral antioxidants have not produced dramatic improvements in conditions associated with redox imbalance, further validation with animal models and clinical trials are necessary to reveal therapeutic potential of combination therapies of antivirals, antioxidant and anti-inflammatory treatments. Understanding the mechanisms that contribute to the pathogenesis of coronavirus infections, will set the foundation for development of new treatments for coronavirus infections.

## Author contributions

CG, SiS, TA, and TK wrote the manuscript, reviewed the literature, and collected the information. SaS revised the manuscript. All authors contributed to the article and approved the submitted version.

## Funding

This work was supported in part by National Institute of Health grant R01AG059501 (TK), National Institute of Health grant R01AG059502 04S1 (TK), and California HIV/AIDS Research Program grant OS17-LA-002 (TK).

## Conflict of interest

The authors declare that the research was conducted in the absence of any commercial or financial relationships that could be construed as a potential conflict of interest.

## Publisher’s note

All claims expressed in this article are solely those of the authors and do not necessarily represent those of their affiliated organizations, or those of the publisher, the editors and the reviewers. Any product that may be evaluated in this article, or claim that may be made by its manufacturer, is not guaranteed or endorsed by the publisher.

## Footnotes

^1^ https://clinicaltrials.gov/
